# Pathways to Care for Young People Diagnosed With Functional Tic Like Behaviours in the United Kingdom: An Online Mixed‐Methods Survey

**DOI:** 10.1111/jpc.70437

**Published:** 2026-05-18

**Authors:** Amanda K. Ludlow, Seonaid Anderson, Sally Robinson, Tamsin Owen, Tammy Hedderly

**Affiliations:** ^1^ Department of Psychology, Sport and Geography University of Hertfordshire Hatfield UK; ^2^ www.neuro‐diverse.org Durbuy Belgium; ^3^ NeuroKids Ltd. London UK; ^4^ TANDeM, Evelina London Children's Hospital, Guy's and St Thomas' London UK; ^5^ Faculty of Life Sciences & Medicine King's College London London UK

## Abstract

**Aim:**

This study aimed to analyse the pathways to obtaining a diagnosis of functional tic‐like behaviour (FTLBs) in the United Kingdom.

**Methods:**

The primary caregivers of children with FTLB (*n* = 55) were recruited through charities and completed an anonymised survey. The authors established the pathway to any diagnosis, gaining information about their child's tics, their impact and the sequence of health‐care professionals (HCPs) they had contacted that had either led or not led to a diagnosis and any support on offer. Mixed‐method analyses were conducted on the surveys.

**Results:**

Many of the characteristics of the children met the criteria for FTLBs, with over half of the children (*n* = 30; 55%) also showing self‐harming and suicidal ideation behaviour. Mothers reported often finding themselves seeing more than three different services and being given differing labels for their child's symptoms (e.g., transient tics and PANDAS). There was little clear guidance on how to manage symptoms, with those who were able to access treatment offered CBT, counselling and/or medication. There were several families still waiting to be seen (*n* = 13; 24%); however, of those who had received a diagnosis, almost one‐third had accessed support from private healthcare providers (*n* = 14; 25%).

**Conclusions:**

Pathways to diagnosis for young people showing FTLBs are complex, time‐consuming and can involve multiple health‐care contacts, with patients alternating between primary and specialised care. Our findings reflect failures in the diagnostic protocols and referral systems of health care in the United Kingdom and a general lack of understanding, specialist skill or knowledge relating to FTLBs.

## 1 Introduction

Around 2020 to 2021, health‐care professionals (HCPs) saw an increase in the severity of tic‐like presentations in those who already had a diagnosis of a tic disorder [1], along with an increase in cases of sudden and new onset severe tics and ‘tic‐like’ attacks [2, 3]. These tic‐like behaviours, now termed functional tic‐like behaviours (FTLBs), appeared to develop and peak in severity within hours to days, causing significant functional impairment, as well as being distressing, painful and hard to manage [4].

The number of cases of FTLBs appears to have decreased since the pandemic, though children and young people with FTLBs are still presenting to clinicians, leading to members of the International MDS Tourette group, the European Society for the Study of Tourette Syndrome (ESSTS) and other professionals to collaborate on producing an international consensus on how to diagnose FTLBs [5], which have been validated in clinical practice [6]. This included (1) patients with FTLBs showing at least one specific complex motor tic (e.g., including complex arm/hand movements and self‐injurious behaviour); (2) having one specific complex phonic tic (e.g., phrases and coprolalia) and (3) having a greater number of complex tics than simple tics, with an age of onset of 12 years or over.

It is recognised that patients presenting with FTLBs can usually be distinguished from those with a primary tic disorder by the speed of tic progression, the clinical course and the type of tics [7]. For example, in FTLBs, motor and vocal tics are usually more complex, involve larger body parts that interfere more with voluntary actions and are less suppressible and more suggestible [8]. They also appear more prominently in females compared to males (the opposite of what is reported in tic disorders), with a much older age of onset [9]. A high co‐occurrence with other conditions is also noted, with Cavanna et al. [10] suggesting that anxiety and functional neurological disorders are commonly associated with functional tics.

While there now appears to be greater clarity around the phenomenology of FTLBs among HCPs, there remains limited research exploring the experiences of families navigating the diagnostic pathway. To address this gap in current understanding, the present study explored the experiences of mothers (only mothers responded) of children with FTLBs in the United Kingdom, addressing the characteristics of the children presenting with FTLBs, the diagnostic process, and support offered to them.

## 2 Methods

Our approach was informed by the STROBE checklist (Data S1) for cross‐sectional studies [11]. Online surveys were developed to elicit parental views and opinions on FTLBs, with data collection running from March 2023 to October 2023. All of the primary caregivers were required to provide written online consent before completing the study.

The online surveys were anonymised and based on diagnostic surveys used for children with autism around pathways to care [12]. They included open‐ended questions on the following:

Details about their child's tics (when and how they first noticed signs, changes in mood/anxiety before or after the tics, particular triggers).

Details about their child's diagnosis (when(if) they got a diagnosis and by whom, any co‐occurring conditions, their medication and whether their children had ever engaged in self‐harm and/or suicidal thoughts).

The impact of tics (how the tics had impacted their child and what they felt were the main challenges).

Management and support for tics (ways in which they were managing the tics, the support they had been offered and advice they would give to other families).

In addition, two standardised measures were chosen: Autism Spectrum Screening Questionnaire (ASSQ; [13, 14]) and Spence Children's Anxiety Scale, parent version (SCAS‐P; [15]).

The ASSQ is a screening tool designed to identify young people who show developmental differences in social and behavioural functioning. It comprises 27 items covering topics regarding social interaction, communication problems and restricted and repetitive behaviour. Total scores range from 0 to 54, with higher scores indicating significant differences from the norm. Whereas the SCAS‐P is a 44‐item questionnaire widely used both clinically and in research to screen for anxiety disorders. The SCAS yields a total score and six sub‐scale scores calculated in line with DSM‐IV anxiety disorder clusters.

Following completion of the study, the primary caregivers were reminded of their right to withdraw and provided with details of where to seek information and support for any concerns around FTLBs.

*Patient and public involvement*: The online questionnaire was developed alongside two mothers of children with FTLBS who did not take part in the final survey. Ethical approval for this research was obtained from the University of Hertfordshire University Health, Science, Engineering and Technology Ethics Committee with Delegated Authority (Protocol number: aLMS/SF/UH/0461D1(1)) and the research was performed in accordance with the Declaration of Helsinki.

### 2.1 Participant Characteristics

A total of 55 mothers (aged *M* = 44.28, SD = 9.28) completed the anonymised online survey. A total of 52 were identified as being white British and three as mixed race. They provided information about their children, of whom there were 35 females, 16 males, 2 non‐binary and 2 transgender males, aged 10 years 6 months–17 years 1 month (*M* = 14.27; SD = 2.56).

On the ASSQ, just over half of children (*N* = 28; 51%) reached the cut‐off scores of 19 or more (*M* = 19.55, SD = 11.73), with mean scores on subscales: Social Interaction (*M* = 12.82, SD = 4.33), Communication (*M* = 4.67, SD = 3.35), Restrictive and Repetitive Behaviours (*M* = 4.38, SD = 2.43), and Motor skills and clumsiness (*M* = 3.5, SD = 2.61). In addition, on the SCAS‐P, almost three quarters of the children (*N* = 39, 71%) were reported to present with symptoms of anxiety that were above the suggested clinical cut‐off (> 24) for clinical diagnosis.

On the SCAS‐P, all participants showed high levels of anxiety across all six subscales: separation anxiety (*M* = 5.86, SD = 4.87), generalised anxiety (*M* = 6.45, SD = 4.00), panic/agoraphobia (*M* = 4.59, SD = 4.27), social phobia (*M* = 9.36, SD = 5.19), obsessive‐compulsiveness (*M* = 4.81, SD = 4.80), physical injury fears (*M* = 4.23, SD = 3.36), with a total (*M* = 37.80, SD = 23.57).

## 3 Results

### 3.1 Neurodevelopmental and Mental Health Needs

**FIGURE 1:**
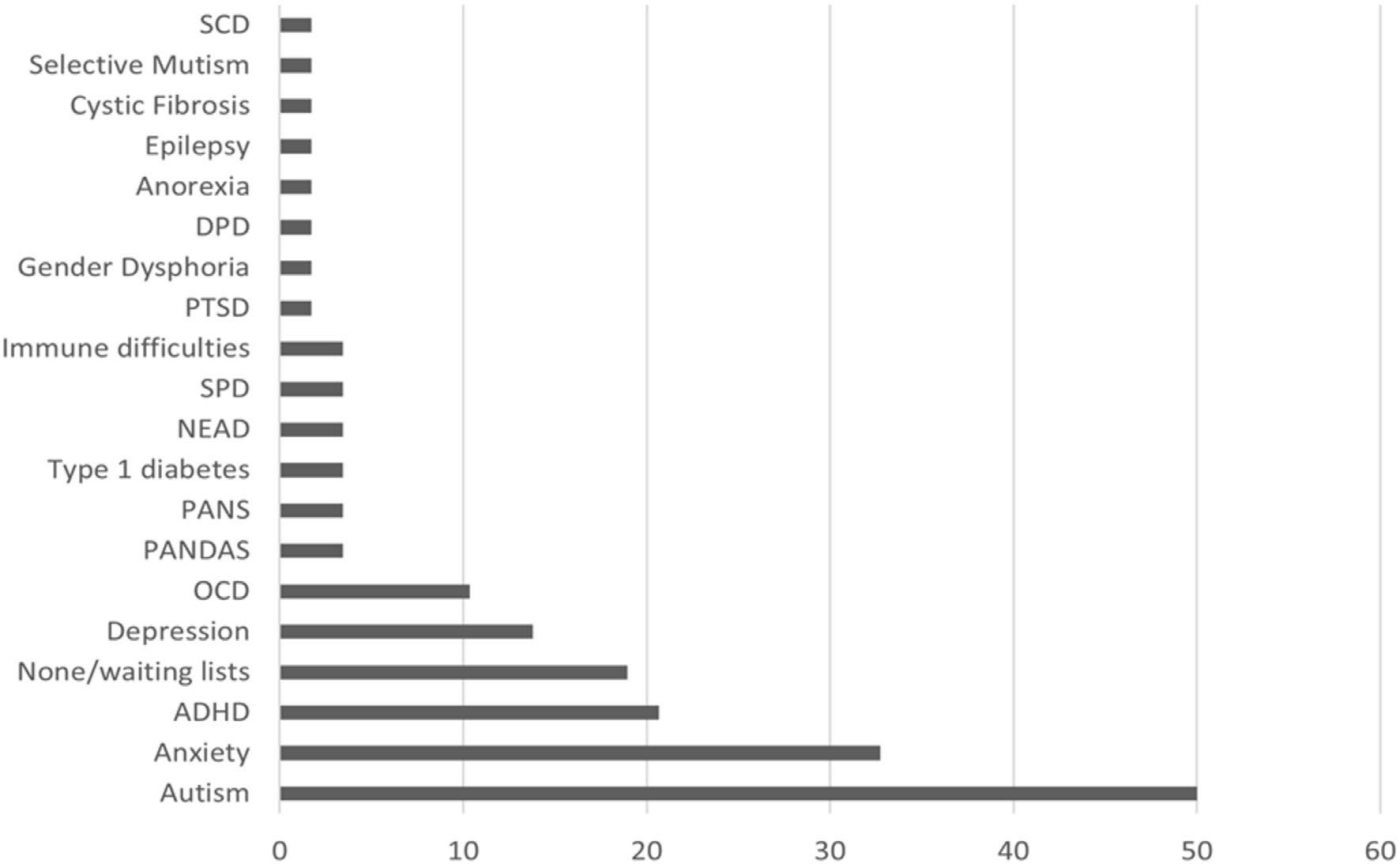
Percentage of young people's co‐occurring diagnoses. Participants could receive more than one diagnosis. ADHD, attention deficit hyperactivity disorder; DPD, dependent personality disorder; NEAD, non‐epileptic attack disorder; OCD, obsessive‐compulsive disorder; PANDAS, paediatric autoimmune neuropsychiatric disorders associated with streptococcal infections; PANS, paediatric acute‐onset neuropsychiatric syndrome; PTSD, post‐traumatic stress disorder; SCD, social communication disorder; SPD, sensory processing disorder.

High levels of co‐occurring conditions were reported across the sample, with 81% of children having one or more additional diagnoses outside of the tics, with a further 9% currently waiting for a referral for autism (*n* = 4) and/or ADHD diagnoses (*n* = 1). The most frequently reported co‐occurring diagnoses included autism (50%), anxiety (33%) and ADHD (21%) (see Figure 1 for additional conditions). Just over half (*n* = 30, 55%) of the children were reported to engage in deliberate self‐harm behaviours and showed signs of suicidal ideation. Notably, two children had already attempted to take their own lives. Similarly, just over half of the children were on medication (51%), including fluoxetine, atomoxetine, clonidine, sertraline, pizotifen, lamictal and melatonin.

### 3.2 Onset and Duration of FTLBs

Many of the primary caregivers (93%) reported tics presenting after 10 years of age (*M* = 12.00, SD = 2.14), with 38% reported after 11 years of age and 55% after 12 years of age. The remaining 7% of children were reported to have experienced the onset of tics between 6 and 10 years of age. All of the primary caregivers reported that the onset of tics began with motor movements—head/neck or shoulder tics. Only 5% (*n* = 3) of the primary caregivers believed that their child's tics would eventually disappear, with half reporting they felt that the tics would persist, as they had already seen a progression in tics with a worsening presentation over time.

### 3.3 Premonitory Urges and Suppression

Most of the primary caregivers (78%) reported that their child felt some sort of sensation and premonitory urge before the tics. This was described as (*an itch*, *a bee sting*, *build‐up in the body*, *a feeling of a shiver without being cold* or *electricity in the neck*). A small minority (22%) reported not being able to suppress the tics, while the remaining children were able to suppress them on occasions for short intervals of time. Resultant behaviours from trying to suppress tics included making them worse, tic attacks, severe exhaustion and headaches.

### 3.4 Presentation of FTLBs

Head and neck jerks were the most reported motor tic (*n* = 31, 56%). Similarly, the most frequent presentation of vocal tics was complex vocalisations (*n* = 28, 51%, e.g., obscene words and phrases atypical of coprolalia in TS), often also presenting with obscene words and gestures (‘coprophenomena‐like’ symptoms). Table 1 provides examples of the types of tics reported, triggers, associated challenges and management strategies.

**TABLE 1:** Examples of the tics, triggers, main challenges and management strategies.

Types of tics	*Motor*: Hitting something (someone, themselves, objects), touching objects/people, throwing objects, neck jerking, whole body jerking, drop attacks, freezing, head turning. Strangling tics, eye rolling, throat clearing, kicking, shoulder shrugs
	*Vocal*: Shouting random, sometimes inappropriate things, swearing, whistling, shouting unkind phrases, talking in different accents
Triggers	Noise, crowded places (shops, school, tiredness, anxiety, OCD, strong emotions)—extremely happy, excited or angry. Being distracted, using screens/gaming increases in demands, overload, sickness and mood
Challenges	All‐encompassing physical and mental exhaustion, physical pain. Impact on their mental health, restricting access to education, unkind treatment at school, leaving the house, public attention and stigma, impact on self‐esteem, self‐harming
Management strategies	Ignore them, talk about it, avoid situations that trigger them, breathing, calming routines (e.g., weighted blankets, going to a quiet room), finding humour, medication, diet

### 3.5 Professionals, Diagnosis and Support

**FIGURE 2:**
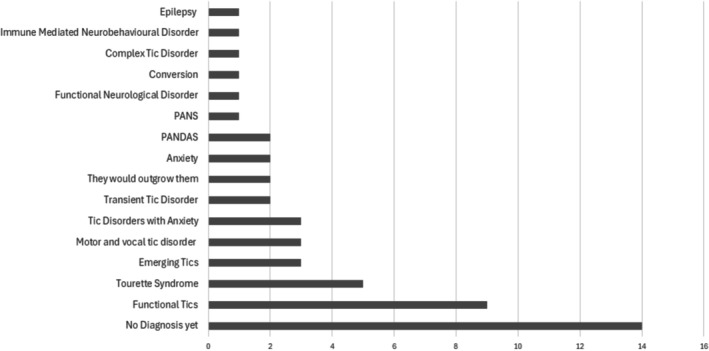
Labels provided by health‐care professionals. Participants could receive more than one diagnosis. PANDAS, paediatric autoimmune neuropsychiatric disorders associated with streptococcal infections; PANS, paediatric acute‐onset neuropsychiatric syndrome.

In total, 76% reported meeting with a HCP. Of whom, half had seen either a paediatrician (25%) or a neurologist (25%); far fewer had seen a psychiatrist, GP and/or clinical psychologist. A total of 24% were still waiting for an appointment. Various labels had been provided to children by HCPs (see Figure 2).

For children who had received a diagnosis, 62% had received no further support, with 14% discharged from the service following diagnosis. The other children had been offered a range of interventions that included medication and/or psychotherapy, cognitive behavioural therapy (CBT), and counselling. Some families had paid for private therapy, often CBT, to try to manage the tics and associated symptoms.

### 3.6 Thematic Data Analysis

Qualitative data from the free‐text responses were analysed using thematic analysis and triangulated with quantitative findings where appropriate. The primary caregivers reported a high level of dissatisfaction with the support provided by HCP. This was reflected in two overarching themes: ‘living with a rare condition’ and ‘importance of management of FTLBSs’. A full list of the themes and sub‐themes is found in Table 2.

Theme 1 Living with a less recognised condition.

**TABLE 2:** Themes and sub‐themes.

Living with a less recognised condition	Minimisation of tics by professionals
	Gap in professionals' understanding
	Lack of a clear referral pathway for FTLBs
Importance of management	Navigating how to manage FTLBs
	Finding your own way through

This theme highlights how a lack of familiarity with FTLBs among HCPs results in the absence of a clear route for diagnosis and/or support for families.

#### 3.6.1 Minimisation of Tics by Professionals

The primary caregivers perceived HCPs as minimising their struggles, with them having to ‘*fight*’ for a referral and the tics attributed to other conditions (e.g., autism, anxiety, PANS)—24% were still waiting to be seen.

During Covid we were referred to CAMHS (urgent referral) and it took over a year to see them. In Sept 22 we saw (Paediatrician) and we were discharged soon after (LS, 51)

We were just told it is something he will grow out of (OW, 48)

#### 3.6.2 Gaps in Professionals' Understanding

Many of the primary caregivers expressed dismay at the diagnostic process, with no clear direction on how to obtain a diagnosis or, in some cases, receiving different diagnoses for the same behaviours.

Nothing. Nobody has a clue what to do and pass us around between professionals. I have been distraught and can't believe the lack of provision (JK, 45)

Initially told anxiety tics. Then told functional tics. Then told Tourettes. Now back to functional tic like behaviour (TC, 46)

We saw numerous GPs, all of whom told us that tics were caused by anxiety and were a normal part of childhood (SW, 45)

#### 3.6.3 A Lack of Clear Referral Pathways for FTLBs

Many of the primary caregivers, including those who chose the private health‐care route, highlighted the need for a ‘*joined‐up*’ system for diagnosis and treatment of FTLBS, with clear care pathways. For example, primary caregivers emphasised the need for their children to be assessed holistically and managed by a team of multidisciplinary specialists (e.g., neurologists, child mental health professionals and speech and language therapists) to fully understand needs and offer appropriate support.

A proper pathway to a correct diagnosis to begin with and then a consistent approach as to what therapy is needed (KD, 35)

To feel listened to and not fobbed off and dismissed and having to self‐refer to everything (SS,49)

Better knowledge by neurologist, better care, better connections to the right therapy (AB, 44)

Theme 2 *Navigating how to manage FTLBs*.

This theme highlighted the need for support in managing FTLBs, with a third of the primary caregivers reporting that no support was offered and having to navigate the health‐care system themselves.

#### 3.6.4 Better Support From HCPs on the Management of FTLBs

A lack of guidance on the management of FTLBs was raised by the majority of the primary caregivers (*n* = 49), and even those who had received a diagnosis highlighted ongoing support as an area of need.

It was a 20‐minute appointment where {child's name} was diagnosed and discharged in the same appointment (SY, 21)

After being sent on a course about FTLBs. The course was useful but didn't say how to manage or deal with them. Some more understanding of how my son could manage would be helpful (RS, 44)

#### 3.6.5 Finding Your Own Way Through

Many mothers reported feeling ‘*abandoned*’ by the medical system and described encouraging others to find their own ways of managing their child's difficulties in the absence of a formal diagnosis.

You are on your own. Do what is right for your child. No one in the medical profession is in the least bit interested in helping you or providing support, advice or answers (IC, 53)

Stay calm and carry on. The child is suffering and needs your support. Don't worry about what others think! (LA, 47)

This difficulty in accessing support led to 30% of the primary caregivers seeking support privately.

We were already paying privately for psychiatrist because waiting time for CAMHS is so long (HG, 50)

If you can afford it, find a knowledgeable private practitioner asap. The NHS won't listen or help (GG, 45)

## 4 Discussion

In the current study, all children appeared to meet the international consensus criteria for FTLBs, although 7% of children reported having a slightly younger age of onset. We found a gender bias towards females, which aligns with previous evidence that FTLBs appear to occur more frequently in females [9]. We also found a high incidence of co‐occurring disorders [6], with over half the children presenting with symptoms within the clinical thresholds for a diagnosis of autism and/or an anxiety disorder.

Our findings also support previously reported phenomenological features characteristic of FTLBs, including complex vocal tic‐like behaviours as well as complex motor behaviours. Three‐quarters of children were reported to experience a premonitory urge before the tic, which was higher than has previously been reported [9]. However, other studies have concluded that this is not a distinguishing factor between individuals with FTLBs with and without a prior history of tics [16].

A range of professionals and services was involved in the assessment of children with FTLBs, reflecting the inconsistency in how services are structured across the United Kingdom. For example, while mental health and neurodisability services are often separate, they typically have distinct referral pathways, offering access to different types of assessment and support. However, this fragmentation of services was reported by the primary caregivers to have contributed to difficulties accessing support for children with FTLBs, with services typically only focusing on a specific set of core symptoms [16]. It is possible that this may have also led to the wide range of diagnostic labels being provided for the FTLBs, suggesting a lack of shared understanding among professionals regarding the diagnosis and management of the condition.

The mental health needs of this group were particularly significant. The primary caregivers described their children experiencing high levels of anxiety and emotional distress, with a high prevalence of suicidal thoughts, plans and attempts—aligning with previous research [17, 18]. It is not clear whether these self‐harming behaviours were related to difficulties in emotional regulation or were secondary to either FTLBs and/or underlying anxiety/depression. However, by exploring emotion regulation strategies, mental health providers and researchers could help lower the risk of self‐harm in young people with FTLBs [19].

Accessing mental health support in the United Kingdom remains challenging, largely due to gaps in service provision and the high clinical thresholds required for entry into many services [20]; however, for children with FTLBs, these challenges appeared to be magnified due to delays in accessing the appropriate pathway and a lack of clarity around which service held responsibility for care. In the absence of integrated assessment and treatment, children were left unsupported at a time of significant clinical need, which, for some families, was reported to contribute to an increase in symptom severity and functional impairment.

There is also much to be learned from those who live with the condition every day, the children's voices. Burn et al. [18], using a qualitative study to address parent' and adolescent' with FTLBs experiences of seeking a diagnosis and post‐diagnostic support, with Oates et al. [21] also adopting a qualitative approach to explore how functional tics affect the young person's daily life, wellbeing, sense of self, help‐seeking, and coping. Both studies have highlighted the challenging journey to gaining support for FTLBs, with being misunderstood by professionals being shown to exacerbate the feelings of distress and loneliness for the young people.

The current findings reinforce the need for clearer care pathways and greater collaboration between mental health and neurodevelopmental services, with integrated service models that provide comprehensive assessment, diagnosis and treatment for children and young people presenting with both neurodevelopmental and mental health needs [22]. Furthermore, there needs to be improved professional awareness and understanding by HCPs in supporting individuals with FTLBs, with access to evidence‐based treatments to improve longer‐term outcomes for these children and their families.

A strength of this study is its inclusion of the primary caregivers' voices, providing valuable insight into their lived experiences of navigating the diagnostic process and accessing support for children with FTLBs. However, future research should ensure both the mothers and fathers are equally represented to help support the effectiveness of the parent‐suggested modifications to service delivery. Furthermore, the online questionnaire was developed alongside two mothers of children with FTLBs, with the selection of the standardised measures (anxiety and autism symptomology) influenced by some previous interviews with mothers [17]. However, it is acknowledged that there are limitations to taking such a targeted approach. Future studies should address other mental health measures beyond anxiety to provide a more comprehensive understanding of mental health and well‐being in young people with FTLBs.

This present study contributes to the existing body of FTLBs by highlighting significant gaps in service provision and understanding among HCPs for the diagnosis and management of FTLBs in the United Kingdom, with the need for clearer care pathways for individuals with FTLBs and often co‐occurring mental health and/or neurodevelopmental needs.

## Funding

The authors have nothing to report.

## Ethics Statement

Ethical approval for this research was obtained from the University of Hertfordshire Health, Science, Engineering and Technology Ethics Committee with Delegated Authority (Protocol Number: aLMS/SF/UH/0461D1(1)) and the research was performed in accordance with the Declaration of Helsinki.

## Consent

All parents provided written consent before completing the study. Upon completion of the survey, parents were reminded of their right to withdraw and provided with details of where to seek information and support for any concerns around FTLBs.

## Conflicts of Interest

The authors declare no conflicts of interest.

## Supporting information


**Data S1:** STROBE Statement—Checklist of items that should be included in reports of cross‐sectional studies.

## Data Availability

The data that support the findings of this study are available from the corresponding author upon reasonable request.
